# Severe Disease in Patients With Recent-Onset Psoriatic Arthritis. Prediction Model Based on Machine Learning

**DOI:** 10.3389/fmed.2022.891863

**Published:** 2022-04-28

**Authors:** Rubén Queiro, Daniel Seoane-Mato, Ana Laiz, Eva Galindez Agirregoikoa, Carlos Montilla, Hye Sang Park, Jose A. Pinto Tasende, Juan José Bethencourt Baute, Beatriz Joven Ibáñez, Elide Toniolo, Julio Ramírez, Cristina Pruenza García-Hinojosa

**Affiliations:** ^1^Faculty of Medicine, Rheumatology Service & the Principality of Asturias Institute for Health Research (ISPA), Universidad de Oviedo, Oviedo, Spain; ^2^Research Unit, Spanish Society of Rheumatology, Madrid, Spain; ^3^Rheumatology and Autoimmune Disease Department, Hospital Universitari de la Santa Creu i Sant Pau, Barcelona, Spain; ^4^Rheumatology Service, Hospital Universitario Basurto, Bilbao, Spain; ^5^Rheumatology Service, Hospital Universitario de Salamanca, Salamanca, Spain; ^6^Rheumatology Service-INIBIC, Complexo Hospitalario Universitario de A Coruña, A Coruña, Spain; ^7^Rheumatology Service, Hospital Universitario de Canarias, Santa Cruz de Tenerife, Spain; ^8^Rheumatology Service, Hospital Universitario 12 de Octubre, Madrid, Spain; ^9^Rheumatology Service, Hospital Universitari Son Llàtzer, Palma, Spain; ^10^Arthritis Unit, Rheumatology Department, Hospital Clínic Barcelona, Barcelona, Spain; ^11^Knowledge Engineering Institute, Universidad Autónoma de Madrid, Madrid, Spain

**Keywords:** recent-onset psoriatic arthritis, severe disease, global pain, perianal psoriasis, prediction model, machine learning

## Abstract

**Objectives:**

To identify patient- and disease-related characteristics that make it possible to predict higher disease severity in recent-onset PsA.

**Methods:**

We performed a multicenter observational prospective study (2-year follow-up, regular annual visits). The study population comprised patients aged ≥ 18 years who fulfilled the CASPAR criteria and less than 2 years since the onset of symptoms. Severe disease was defined at each visit as fulfillment of at least 1 of the following criteria: need for systemic treatment, Health Assessment Questionnaire (HAQ) > 0.5, polyarthritis. The dataset contained data for the independent variables from the baseline visit and follow-up visit number 1. These were matched with the outcome measures from follow-up visits 1 and 2, respectively. We trained a logistic regression model and random forest–type and XGBoost machine learning algorithms to analyze the association between the outcome measure and the variables selected in the bivariate analysis.

**Results:**

The sample comprised 158 patients. At the first follow-up visit, 78.2% of the patients who attended the clinic had severe disease. This percentage decreased to 76.4% at the second visit. The variables predicting severe disease were patient global pain, treatment with synthetic DMARDs, clinical form at diagnosis, high CRP, arterial hypertension, and psoriasis affecting the gluteal cleft and/or perianal area. The mean values of the measures of validity of the machine learning algorithms were all ≥ 80%.

**Conclusion:**

Our prediction model of severe disease advocates rigorous control of pain and inflammation, also addressing cardiometabolic comorbidities, in addition to actively searching for hidden psoriasis.

## Introduction

Psoriasis is a chronic skin disease that affects 2–3% of the general population in developed countries. The condition most frequently associated with psoriasis is psoriatic arthritis (PsA), which affects approximately one third of affected patients. Both diseases constitute the main poles of what we today know as psoriatic disease, a systemic entity in which joint and skin manifestations are accompanied by manifestations that are typical of spondylarthritis, such as uveitis and inflammatory bowel disease, together with a wide range of comorbid conditions, the most relevant of which for the final prognosis of PsA are cardiovascular conditions (1).

Therefore, the concept of severity in psoriasis is wide-ranging and can be envisaged from different perspectives. Severity can refer mainly to the inflammatory burden of the disease and its most immediate consequences, such as loss of physical function and structural damage (the latter appearing more in the medium and long terms), or extend to quality of life, and even vital prognosis. In this sense, we have known for decades that patients with greater inflammatory burden and structural damage (erosions) also have the highest risk of mortality, which, in this population, is almost always cardiovascular in origin (1, 2). Consequently, the concept of severity in PsA associates inflammatory burden with structural damage and increased mortality. In other words, it would be necessary to extend the concept of severity beyond its most immediate significance (inflammation and loss of function) so that it covers vital prognosis, which is often shortened owing to the co-occurrence of cardiovascular events (1–3).

With respect to daily clinical practice, it is essential to know which characteristics of PsA predict higher “immediate” disease severity (i.e., reversible severity), understood as more pronounced inflammatory activity, together with greater functional limitation. This would make it possible to exert a positive influence on the other “long-term” severity (i.e., irreversible severity), which is more closely associated with co-occurrence of cardiometabolic comorbid conditions, cumulative structural damage, and poorer survival, all of which represent no more than poor long-term control of “immediate” severity. The objective of the present prospective study was to identify disease- and patient-related characteristics that predict higher “immediate” severity in recent-onset PsA.

## Materials and Methods

The design of the REAPSER study has been described in detail elsewhere (4).

REAPSER is a multicenter observational prospective study (2-year follow-up, regular annual visits), promoted by the Spanish Society of Rheumatology. The study population comprised patients of both sexes aged ≥ 18 years who fulfilled the Classification Criteria for Psoriatic Arthritis (CASPAR) (5), with less than 2 years since the onset of symptoms attributable to the disease.

The intention at the baseline visit was to reflect the patient’s situation before disease progress was modified by the treatments prescribed in the rheumatology department. In this sense, participants could not have been receiving methotrexate, leflunomide, or apremilast for more than 3 weeks after initiation and could not be receiving biologic disease-modifying antirheumatic drugs (DMARDs). These intervals were fixed considering that the mean time from initiation of treatment until onset of the response to therapy is 4 weeks in the case of synthetic DMARDs and 1 week in the case of biologic DMARDs. In cases where the patient had been receiving synthetic DMARDs for more than 3 weeks, we obtained confirmation from the investigating rheumatologist that the patient had not yet responded to treatment at the baseline visit; this information was sought in only 9 patients, and for all those involved, the time since initiation of synthetic DMARDs was under 2 months.

If patients with psoriasis receiving treatment with synthetic or biologic DMARDs developed PsA and were referred to the rheumatology department for diagnosis and management, then they could be included in the study, since this would not violate the criterion that the baseline visit reflected the situation of the patient before disease progress was modified by the treatment prescribed at the rheumatology clinic.

Patients were invited to participate consecutively at one of their scheduled visits to the rheumatologist. Recruitment began in November 2014 and ended in October 2016. A total of 25 centers from 11 of the 17 Spanish autonomous communities participated in the study.

All patients gave their informed consent to participate. The study centers assigned each participant an identification code in order to ensure data confidentiality in line with current legislation. The study complies with the Declaration of Helsinki and was approved by the Clinical Research Ethics Committees of the Principality of Asturias (study number 14/2014).

### Variables and Measurement

a) Sociodemographic data: age; sex; educational level (none, primary, secondary, university).
b) Family history (father, mother, grandparents, siblings, children) of PsA, other types of inflammatory arthritis, and psoriasis.
c) Personal history and comorbidities (based on a review of medical records): age-adjusted Charlson comorbidity index (6), cardiovascular risk factors [arterial hypertension, hyperlipidemia, diabetes mellitus (differentiating between insulin- and non–insulin-dependent)].
d) Anthropometric data: body mass index (BMI).
e) Lifestyle: smoking (patients who reported having smoked at least 100 cigarettes in their lifetime and who at the time of the visit smoked every day or on some days were classified as “current smoker.” Patients who reported having smoked at least 100 cigarettes throughout their lifetime and who at the time of the visit did not smoke at all were classified as “ex-smokers.” Patients who reported not having smoked 100 cigarettes were defined as “never smokers”). Alcohol consumption was measured in standard alcohol units per week and evaluated using the Systematic Interview of Alcohol Consumption (7). Physical activity was evaluated using the short form of the International Physical Activity Questionnaire (IPAQ); 3 levels were established for the analysis (low, moderate, and high), according to the guidelines for data processing and analysis of the IPAQ (8).
f) Clinical situation at diagnosis of PsA: year of presentation of symptoms of PsA; clinical form [(1) axial, (2) peripheral, (3) mixed]; articular pattern [(1) oligoarticular, (2) polyarticular, (3) distal, (4) mutilans, (5) spondylitis]; presence of dactylitis (yes/no).
g) Joint involvement and enthesitis: number of tender joints (NTJ68); number of swollen joints (NSJ66); extended version of the Maastricht Ankylosing Spondylitis Enthesitis Score (MASES) (9). Polyarthritis was defined as NSJ66 ≥ 5.
h) Pain and global assessment of disease during the previous week: Patient global pain on a scale ranging from 0 (no pain) to 10 (very intense); patient global assessment of disease on a scale ranging from 0 (feels very well) to 10 (feels very ill); physician global assessment of disease on a scale ranging from 0 (minimal activity) to 10 (maximum activity).
i) Cutaneous and nail involvement (evaluated by a dermatologist): cutaneous psoriasis (yes/no); year of onset of psoriasis; clinical type [psoriasis vulgaris (plaques), guttate, erythrodermic, generalized pustular, localized pustular, inverse, other]; specific locations (scalp, nails, palms and soles, gluteal cleft and/or perianal region, palmoplantar pustulosis, mucosal involvement); treatment of psoriasis and year of onset (topical treatment, phototherapy, retinoids, methotrexate, cyclosporine, etanercept, infliximab, adalimumab, ustekinumab, other). Body surface area (BSA) affected by psoriasis or Psoriasis Area and Severity Index (PASI) (10); onychopathy (number of digits affected). For purposes of the analysis, severe psoriasis was defined as PASI > 10.
j) Functional situation and quality of life: Health Assessment Questionnaire (HAQ) (11), Psoriatic Arthritis Impact of Disease (PsAID) (12).
k) Radiographic evaluation at baseline: Bath Ankylosing Spondylitis Radiology Index (BASRI) of the sacroiliac region (13), hand involvement according to the modified Steinbrocker method for PsA (14).
l) Laboratory tests: C-reactive protein (CRP), uric acid, total cholesterol, LDL cholesterol, triglycerides. For purposes of the analysis, a series of cut-off points were established to define high values: > 0.5 mg/dl for standard CRP; > 0.3 mg/dl for high-sensitivity CRP; hyperuricemia if > 7 mg/dl in men and > 6 mg/dl in women; ≥ 200 mg/dl for total cholesterol; ≥ 100 mg/dl for LDL; ≥ 150 mg/dl for triglycerides.
m) Treatment of PsA with DMARDs, date of initiation, date of finalization: synthetic DMARDs (methotrexate, leflunomide, sulfasalazine, apremilast, cyclosporine), biologic DMARDs (adalimumab, etanercept, infliximab, golimumab, ustekinumab, certolizumab, secukinumab).

Severe disease was defined at each visit as fulfillment of at least 1 of the following criteria: treatment with DMARDs, HAQ > 0.5, polyarthritis. Investigators didn’t know this definition when assessing the patients.

### Sample Size

REAPSER study was planned as a registry intended to collect a large number of variables, without prespecified hypothesis. The initial estimation for recruitment in the REAPSER cohort was 295 patients, assuming that up to 25% could be lost to follow-up. This sample size would make it possible to detect as significant a relative risk > 2.30, assuming an exposure of 50% (conservative assumption to maximize the required sample size), confidence level of 95%, and statistical power of 80%.

### Statistical Analysis

#### Imputation of Missing Data

- The duration of psoriasis was imputed with the median of the remaining patients from the same age range. The age ranges used were as follows: < 41 years, 41–60 years, and > 60 years.
- Systemic treatment of psoriasis was imputed with 0 (that is, not receiving systemic treatment).
- Radiological involvement of the hands at the baseline visit was not imputed, except for those patients with an NTJ28 and NSJ28 value of 0, in which case it was imputed with 0.
- For patients who stopped attending the visits owing to improvement of their condition, the missing values for the variables PsAID, HAQ and severe disease were imputed with 0.

#### Generation of the Dataset

The analysis was performed to determine predictive ability, attempting to establish associations between the outcome measures and values at the previous visit for the remaining variables. To do so, the dataset contained data for the independent variables from the baseline visit and from follow-up visit number 1. These were matched with the outcome measures from follow-up visits 1 and 2, respectively. Atemporal variables such as sex and family history were matched with outcome measures from follow-up visits 1 and 2; therefore, their values are the same for each one. This was also true for variables that were only collected at the baseline visit, such as systemic treatment of psoriasis at PsA diagnosis and clinical form at diagnosis.

#### Bivariate Analysis

We selected variables whose Spearman correlation was considered significant according to the threshold applied to the ρ correlation coefficient ($\left|p\right|>\frac{2}{\sqrt{N}}$, with N being the number of data items). We also applied methods based on artificial intelligence, specifically the XGBoost algorithm and the SHAP technique, in order to identify informative variables (see Supplementary Material for a detailed explanation of both approaches). Finally, of the variables identified in the previous steps, we selected those that were statistically significantly associated with the outcome measure (*p* < 0.05). To do so, we applied the Mann–Whitney test for continuous/discrete variables and the χ^2^ test for categorical variables.

#### Multivariate Analysis

In order to generate models where the independent variables do not share information, we selected statistically significant variables (i.e., *p* < 0.05) in an iterative fashion. The steps were performed in the 75% of the sample (training dataset) as follows:

• A logistic regression model based on artificial intelligence was trained with all the variables selected in the bivariate analysis, and the *p*-values of the Wald tests were obtained for each variable (the null hypothesis of this test is that there would be no statistically significant differences in the functioning of the model if the coefficient of the variable was zero, that is, if it was not associated with the outcome measure).
• The variable with the highest *p*-value (Wald test) was withdrawn.
• An additional logistic regression model was trained with the rest of variables.
• These steps were repeated until no variable passed the Wald test (*p* < 0.05).

Next, based on the variables selected using the previous steps, random forest–type and XGBoost machine learning algorithms were trained to analyze the association between the outcome measure and the variables selected (see Supplementary Material for more detail). To train the machine learning models the sample is split in two subsets, one to train the model and the other to evaluate its functioning. The division is generated in such a way that the proportion for each class of the outcome measure is the same in both subsets.

When the subsamples generated are imbalanced, the oversampling technique is used to train the models. This is based on duplicating or triplicating those data whose value for the outcome variable is a minority value.

The parameters and thus the predictions of the trained algorithm might depend on the randomness that derives from the train/test split, which means that different splits of the data might result in different models. To reduce this effect, k-fold cross-validation was performed. Such method consists in splitting the original dataset into k subsets of the same size, and iteratively training the algorithm with k-1 of them while testing the model with the one left. After k iterations, the algorithm will have been trained and evaluated with all the partitions. In this analysis, a k-fold cross-validation with *k* = 5 has been used for both the random forest and XGBoost. Hence, the models were trained with 80% of the data at each iteration, while their good functioning was evaluated with the remaining 20% of the data. The subsets used were the same for the random forest and XGBoost.

The contribution of the variables to the prediction of each algorithm was calculated by the feature importance of each variable in the training data. To estimate the performance of the algorithms we calculated the mean of the values of accuracy, sensitivity, specificity, positive predictive value, and negative predictive value in the different evaluations performed in the cross validation.

## Results

The sample eventually comprised 158 patients. Table 1 summarizes the baseline characteristics.

**TABLE 1:** Baseline characteristics of the sample.

Variable	
Age	49.35 (13.53)
Sex	
Male	90 (57%)
Female	68 (43%)
Educational level	
None	3 (1.9%)
Primary	58 (36.7%)
Secondary	66 (41.8%)
University	31 (19.6%)
BMI	27.63 (5.27)
Smoking	
Never smoked	61 (38.6%)
Exsmoker	44 (27.8%)
Occasional smoker	6 (3.8%)
Daily smoker	47 (29.7%)
Weekly alcohol consumption	0 (0–4)
Family history of psoriasis	62 (39.2%)
Family history of psoriatic arthritis and other types of inflammatory arthritis	21 (13.3%)
Age-adjusted Charlson comorbidity index	1 (0–2)
Arterial hypertension	39 (24.7%)
Hyperlipidemia	53 (33.5%)
Diabetes mellitus	
Non–insulin-dependent	13 (8.2%)
Insulin-dependent	3 (1.9%)
Psoriasis	149 (94.3%)
Duration of psoriasis until onset of PsA (years)	10 (2–20)
Clinical form of psoriasis	
Vulgaris	126 (80.3%)
Guttate	5 (3.2%)
Localized pustular	10 (6.4%)
Inverse	7 (4.5%)
Psoriasis specific sites	
Scalp	88 (59.5%)
Nails	91 (61.5%)
Palms and soles	13 (8.8%)
Gluteal cleft and/or perianal region	34 (23.0%)
Mucous membranes	1 (0.7%)
PASI	1.2 (0.3–3.1)
Systemic treatment of psoriasis	21 (14.3%)
Clinical form of PsA	
Axial	12 (7.6%)
Peripheral	126 (79.7%)
Mixed	20 (12.7%)
Main joint pattern in PsA	
Oligoarticular	87 (55.1%)
Polyarticular	47 (29.7%)
Distal	9 (5.7%)
Spondylitis	15 (9.5%)
Dactylitis at diagnosis	71 (44.9%)
Enthesitis at diagnosis	43 (27.2%)
Uveitis at diagnosis	1 (0.6%)
Pain in the previous week	5 (3–7)
Patient global assessment of disease	5 (3–7)
PsAID	3.75 (1.65–5.90)
Sacroiliac involvement (BASRI)	0 (0–1)
Hand involvement (modified Steinbrocker)	0 (0–2)

*Categorical variables are expressed as n (%). Numerical variables are expressed as mean (SD) if normally distributed and as median (IQR) if not.*

Thirty-three patients (20.9%) were lost to follow-up. The investigating rheumatologist at their center confirmed that 10 of these patients had not attended the visit because their PsA had improved.

At the first follow-up visit, 78.2% of the patients who attended the clinic had severe disease. This percentage decreased to 76.4% at the second visit.

### Bivariate Analysis

Table 2 shows the variables selected in the bivariate analysis.

**TABLE 2:** Variables associated with severe disease. Bivariate analysis.

Variable	*p*-value
Clinical form at diagnosis	< 0.001
High CRP	0.01
Diabetes	0.02
Arterial hypertension	0.001
Psoriasis affecting the gluteal cleft and/or perianal region	0.04
Swollen joint count	< 0.001
Tender joint count	< 0.001
Treatment with synthetic DMARDs	< 0.001
Physician global assessment of disease	< 0.001
Patient global assessment of disease	< 0.001
Patient global pain	< 0.001
PsAID	< 0.001
HAQ	< 0.001

### Multivariate Analysis

The number of observations for the multivariate analysis was 364.

Given that all patients with diabetes had severe disease, it was necessary to apply an L1 regularization in order to assign a coefficient in the logistic regression analysis. The regularization limits the magnitude of the regression coefficients so that the model can generalize for new data. In this case, given that all patients with diabetes had severe disease in the training data, the model run without regularization assigned coefficients of +∞ to the variable diabetes, in such a way that if a patient had diabetes, he/she would be always classified as severe disease. L1 regularization limits the coefficient of the variable diabetes so that the model envisages the case of a patient with diabetes having non-severe disease. In mathematical terms, coefficients are limited by adding the sum of the absolute values of the coefficients to the error function, which, in turn, when minimized reveals the coefficients.

Table 3 shows the results of the logistic regression analysis. The variables predicting severe disease in PsA at the following visit and selected in this analysis were patient global pain, clinical form at diagnosis, psoriasis affecting the gluteal cleft and/or perianal area, treatment with synthetic DMARDs, arterial hypertension, and high CRP. The direction of the association was positive for all 6 variables.

**TABLE 3:** Variables associated with severe disease selected in the logistic regression analysis.

Variable	Regression coefficient	95% CI	*p*-value (Wald test)
Patient global pain	5.174	(3.447, 6.901)	< 0.001
Clinical form at diagnosis	4.490	(2.157, 6.822)	< 0.001
Psoriasis affecting the gluteal cleft and/or perianal area	2.270	(0.945, 3.595)	0.001
Treatment with synthetic DMARDs	2.177	(1.393, 2.961)	< 0.001
Arterial hypertension	1.918	(0.864, 2.972)	< 0.001
High CRP	1.568	(0.662, 2.474)	0.001

When the random forest–type and XGBoost machine learning algorithms were trained with these six variables, patient global pain and treatment with synthetic DMARDs were the most important variables according to the values of feature importances (Table 4).

**TABLE 4:** Feature importances of the variables in the different models trained in the cross validation.

Variable	Iteration 1	Iteration 2	Iteration 3	Iteration 4	Iteration 5
**Random Forest**					
Patient global pain	0.455	0.354	0.428	0.354	0.453
Treatment with synthetic DMARDs	0.236	0.348	0.296	0.348	0.292
Clinical form at diagnosis	0.089	0.099	0.066	0.079	0.071
High CRP	0.078	0.059	0.073	0.063	0.062
Arterial hypertension	0.072	0.077	0.060	0.078	0.063
Psoriasis affecting the gluteal cleft and/or perianal area	0.071	0.063	0.078	0.078	0.060
**XGBoost**					
Treatment with synthetic DMARDs	0.491	0.544	0.601	0.426	0.531
Patient global pain	0.119	0.131	0.096	0.123	0.126
Clinical form at diagnosis	0.079	0.090	0.040	0.142	0.048
High CRP	0.094	0.074	0.095	0.092	0.089
Arterial hypertension	0.098	0.091	0.086	0.112	0.083
Psoriasis affecting the gluteal cleft and/or perianal area	0.119	0.070	0.082	0.106	0.123

Table 5 shows the mean of the values of accuracy, sensitivity, specificity, positive predictive value, and negative predictive value in the different evaluations performed in the cross validation.

**TABLE 5:** Measures of validity in the different evaluations performed in the cross validation.

Metric	Accuracy	Sensitivity	Specificity	NPV	PPV
**Random Forest**					
**Mean**	83.75	81.24	86.18	82.50	86.63
**SD**	4.69	3.53	11.44	1.71	9.79
**95% CI**	70.72, 96.78	71.45, 91.03	54.41, 100.00	77.74, 87.25	59.44, 100.00
**XGBoost**					
**Mean**	82.20	80.72	83.65	81.43	83.35
**SD**	7.10	5.60	9.70	5.62	8.92
**95% CI**	62.50, 100.00	65.18, 96.25	56.72, 100.00	65.83, 97.03	58.59, 100.00

*SD, standard deviation.*

## Discussion

In this multicenter prospective study carried out in patients with recent-onset PsA, assessed at baseline before the potential modification of its natural history because of the treatment prescribed by a rheumatologist, an artificial intelligence–based analysis revealed 6 variables that could predict severe disease on the following year’s visit: pain, treatment with synthetic DMARDs, clinical form at diagnosis, high CRP value, arterial hypertension, and psoriasis affecting the gluteal cleft and/or perianal area. The mean values of the measures of validity of the machine learning algorithms were all ≥ 80%.

Pain and the use of synthetic DMARDs were the most important variables in the predictive hierarchy generated by our models. The importance of the other four variables was similar between them.

Pain is the variable to which patients traditionally attribute the greatest weight and relevance in questionnaires on disease impact and activity. For example, pain is one of the components of DAPSA sum score (15); moreover, in the PsAID questionnaire, pain is the item that receives the greatest weighting, thus increasing its contribution to the final PsAID score to 3 times the VAS score for pain (12). In any case, we must ask why joint pain can predict higher severity, as defined in the present study. Importantly, a significant percentage of patients with psoriasis or PsA who experience pain present with inflammatory activity that can only be detected by means of sensitive imaging techniques (high-resolution ultrasound or magnetic resonance), even if this activity is not apparent in the physical examination (subclinical inflammation) (16). Furthermore, when patients with an oligoarticular pattern (≤ 4 inflamed joints) are examined using sensitive imaging techniques, many are reclassified to a polyarticular pattern (≥ 5 inflamed joints) (16). Detection of this type of subclinical inflammation by adequate imaging techniques is a short-term predictor (6 months) of clinically evident flare-ups (16). In summary, an increase in the intensity of pain or in the tender joint count could be considered, in specific circumstances (excluding non-inflammatory forms of pain, such as fibromyalgia) to be a surrogate marker of greater inflammatory activity. However, these findings should be confirmed using sensitive imaging techniques. This is not a minor issue, since we have evidence that some drugs approved for the treatment of psoriasis and PsA can reverse this subclinical inflammation (16).

The use of synthetic DMARDs was associated with more severe disease at subsequent visits. This seems obvious, since patients with more active PsA are more prone to receive these agents early, and cases with a greater inflammatory burden (≥ 5 joints affected) have, in turn, traditionally been associated with more severe disease and worse prognosis (17). Therefore, this first explanation is no more than a type of circular reasoning. However, an alternative reading is also possible. We know that in other diseases, conventional DMARDs are independently associated with lower odds of achieving treatment goals (18). If a similar situation is occurring in PsA, this should be assessed in larger-scale studies. In our sample, 8.8% of patients receiving conventional DMARDs had polyarthritis in their visit the following year compared with 4.1% of patients who were not receiving conventional DMARDs. We also know that treat-to-target strategies are associated with higher odds of successful therapy and that almost all involve early introduction of biologics (19). A practical consequence of this finding is that we should not rule out a strategy based on early introduction of biologics or targeted therapy in patients with PsA and factors related to severity or poor prognosis.

Our analysis revealed mixed forms (axial and peripheral) at diagnosis as being associated with greater severity. It is biologically plausible that patients with both axial and peripheral involvement not only experience greater inflammatory burden owing to the extension of the skeletal segments involved, but that they also develop greater physical disability, which would be detected using the HAQ-Disability Index, one of the elements included in the *ad hoc* definition of severity in the present study. In fact, several studies confirm that patients with PsA and axial involvement have more severe psoriasis, a greater number of tender peripheral joints, poor physical function, and lower quality of life (20). Obesity, which has been associated with increased activity and severity of PsA, poorer retention of biologics, and reduced possibilities of achieving minimal disease activity, seems to entail a greater risk of axial involvement (21). Our findings have a clear practical application since not all drugs administered to treat PsA cover equally well the axial domain of the disease. In fact, the only specific randomized clinical trial on this domain has been with secukinumab (22).

Increased biological activity measured based on high inflammatory biomarker levels such as CRP, has traditionally been considered an indicator of poor prognosis, although it has also been considered to predict a good response to biological therapy. In the EULAR recommendations for PsA management, persistently elevated acute phase reactant values are associated with severity (23). Therefore, our findings simply corroborate this already classic finding.

The last 2 characteristics detected by our artificial intelligence algorithm as predictive factors are somewhat more complex to understand, since, to our knowledge, it is the first time such an association has been established. Hypertension is a component of metabolic syndrome, and we know that the prevalence of the syndrome and of its individual components is higher in patients with psoriatic disease than in the general population, even more so than in other chronic conditions such as rheumatoid arthritis and axial spondyloarthritis (3). When PsA is compared with psoriasis alone, hypertension is more prevalent in the former (24). Other elements of metabolic syndrome are also more prevalent in PsA than in psoriasis alone (3). These findings can be attributed to an aberrant over-expression of interleukin 17A, a cytokine with a key role in the pathogenesis of psoriatic disease, which also seems to be crucial in the pathogenesis of metabolic syndrome, since it is clearly associated with insulin resistance and desensitization of insulin receptors in the liver and in muscle tissue. This and other proinflammatory cytokines in adipose, liver, and muscle tissue are responsible for a low-grade proinflammatory state known as adipo-inflammation or metabolic inflammation. This type of inflammation is more common in PsA than in other inflammatory diseases (25). An indirect proof of this association was recently seen with the publication of 2 studies based on clinical practice. In a multicenter Italian study, the authors found that obese patients with PsA had higher blood levels of IL17A than patients with normal weight and that this was in turn associated with a better response to secukinumab, a direct IL17A inhibitor, in obese patients (26). Moreover, a Spanish multicenter study showed that the variables associated with better survival of secukinumab in PsA and axial spondyloarthritis, apart from male sex, were obesity, hypertension, and diabetes (27). Furthermore, IL17A is increasingly showing a direct pathogenic role in obesity (3), diabetes (3), and hypertension (28). Therefore, our finding of a predictive association between hypertension and disease severity should be considered in this IL17-dependent setting.

Finally, we found an association between a specific area affected by psoriasis and a higher risk of severe disease. We have known for at least a decade that psoriasis affecting gluteal cleft and/or perianal area is associated with a greater risk of PsA (29). Several hypotheses can be formulated to explain the association with severe disease. First, these areas are difficult to treat and are very uncomfortable for the patient; therefore, the physician may be more likely to treat them more actively, with early prescription of systemic treatment. We must remember that one of the criteria for severe disease in the present study was the use of systemic treatment. Second, a topographical affinity could be established between this affected area and, for example, a greater degree of sacroiliac joint involvement. As stated earlier, involvement of the axial skeleton seems to increase disease severity. A topographical affinity has traditionally been established between psoriasis of the scalp and involvement of the cervical column (30). Nevertheless, plausible biological explanations for this affinity have not been established. Finally, we could speculate as to the biological uniqueness of this affected site, which, owing to mechanisms that have not yet been elucidated, may be associated not only with a greater risk of PsA, but also with greater severity. Therefore, further research is necessary.

The main limitation of this study is its sample size and the fact that some data are missing for some variables. This affected the power of the statistical analysis and, therefore, the ability of the study to detect variables associated with the outcome measure. We tried to compensate for this by using models based on artificial intelligence and machine learning. Random forests are “joint” algorithms in which decision trees are trained with different subsets of variables and data. Decision trees are more flexible than many statistical models, since they make it possible to identify many types of association between explanatory variables and the outcome measure. Furthermore, the fact that random forests add variability prevents the model from being overadjusted to the data and can be re-run with new data, thus increasing the robustness of the predictions. On the other hand, XGBoost algorithms use ensembles of decision trees in a sequential manner. In each tree, the observations that were wrongly classified in the previous one are given a larger weight, thus creating models with very little bias which usually result in very accurate predictions. The counterpart of this phenomenon is a higher risk of the model being overfit to the training dataset. Our analysis showed that the random forest models tended to perform better than XGBoost in terms of all the metrics, which is probably due to the reduced number of observations in the dataset causing the training and test subsets to be quite disparate. Therefore, we could conclude that for such small datasets, an algorithm that overfits less to the training subset such as random forest is more appropriate.

The main strength of this study is its ability to record the course of PsA from an early phase before the natural disease evolution is modified by treatment prescribed by the rheumatologist.

## Conclusion

Taken together, our results could guide clinical practice and management of severe PsA through the following:

- Rigorous strategies for control of pain and inflammation
- Active search for occult psoriasis in the gluteal cleft and perianal area
- Active search for axial involvement and management thereof in line with the summary of product characteristics of the drugs indicated for this domain if the patient’s clinical circumstances so require
- Active search for subclinical enthesitis or synovitis with sensitive imaging techniques in patients with PsA and increasing pain
- Control of cardiometabolic factors in line with current evidence and based on a pathogenesis common to these factors and PsA.

## Data Availability Statement

The raw data supporting the conclusions of this article will be made available by the authors, without undue reservation.

## Ethics Statement

The studies involving human participants were reviewed and approved by the Clinical Research Ethics Committees of the Principality of Asturias (study number 14/2014). The patients/participants provided their written informed consent to participate in this study.

## Author Contributions

RQ participated in the design of the work, the acquisition and interpretation of data, and was a major contributor in writing the manuscript. DS-M participated in the design of the work, the analysis and interpretation of data, and was a major contributor in writing the manuscript. AL, EG, CM, HP, JP, JB, BJ, ET, and JR made substantial contributions to the acquisition of data. CP participated in the analysis of data. All authors revised the manuscript critically, and read and approved the final version.

## Conflict of Interest

DS-M received honoraria from Galapagos for an educational event. AL received payment or honoraria for speakers’ bureaus and educational events, support for attending meetings, and participation on Advisory Boards from Novartis, Pfizer, Amgen, Janssen, and Lilly. EG received payment for presentations, support for attending meetings, and participation on Advisory Boards from Novartis, Pfizer, Amgen, Janssen, Lilly, AbbVie, MSD, Roche, and UCB. JP received payment for presentations, support for attending meetings, and participation on Advisory Boards from Janssen, Novartis, and Lilly. JB received payment for a presentation from Amgen and support for attending meetings from AbbVie and Pfizer. BJ received payment for speaker bureau, support for attending meetings, and participation on Advisory Boards from Novartis, UCB, and Amgen. JR received consulting fees, payment for presentations, support for attending meetings, and participation on Advisory Boards from MSD, Novartis, AbbVie, Pfizer, Janssen, Amgen, UCB, and Lilly. The remaining authors declare that the research was conducted in the absence of any commercial or financial relationships that could be construed as a potential conflict of interest.

## Publisher’s Note

All claims expressed in this article are solely those of the authors and do not necessarily represent those of their affiliated organizations, or those of the publisher, the editors and the reviewers. Any product that may be evaluated in this article, or claim that may be made by its manufacturer, is not guaranteed or endorsed by the publisher.
